# Prognostic Value of microRNA-221/2 and 17-92 Families in Primary Glioblastoma Patients Treated with Postoperative Radiotherapy

**DOI:** 10.3390/ijms22062960

**Published:** 2021-03-15

**Authors:** Elena Schnabel, Maximilian Knoll, Christian Schwager, Rolf Warta, Andreas Mock, Benito Campos, Laila König, Christine Jungk, Wolfgang Wick, Andreas Unterberg, Jürgen Debus, Christel Herold-Mende, Amir Abdollahi

**Affiliations:** 1 German Cancer Consortium (DKTK) Core-Center, German Cancer Research Center (DKFZ), 69120 Heidelberg, Germany; elena.schnabel@med.uni-heidelberg.de (E.S.); m.knoll@dkfz.de (M.K.); c.schwager@dkfz.de (C.S.); andreas.mock@med.uni-heidelberg.de (A.M.); Laila.Koenig@med.uni-heidelberg.de (L.K.); juergen.debus@med.uni-heidelberg.de (J.D.); 2 Heidelberg Ion-Beam Therapy Center (HIT), Divisions of Molecular & Translational Radiation Oncology, Heidelberg University Hospital (UKHD), 69120 Heidelberg, Germany; 3 National Center for Radiation Research in Oncology (NCRO), Heidelberg Institute of Radiation Oncology (HIRO), DKFZ and UKHD, 69120 Heidelberg, Germany; 4 CCU Translational Radiation Oncology, National Center for Tumor Diseases (NCT), DKFZ and UKHD, 69120 Heidelberg, Germany; 5 Center for Child and Adolescent Medicine, General Pediatrics, Heidelberg University Hospital, 69120 Heidelberg, Germany; 6 Division of Experimental Neurosurgery, Department of Neurosurgery, Heidelberg University Hospital, 69120 Heidelberg, Germany; rolf.warta@med.uni-heidelberg.de (R.W.); benito.campos@med.uni-heidelberg.de (B.C.); christine.jungk@med.uni-heidelberg.de (C.J.); andreas.unterberg@med.uni-heidelberg.de (A.U.); h.mende@med.uni-heidelberg.de (C.H.-M.); 7 National Center for Tumor Diseases (NCT) Heidelberg, Department of Medical Oncology, Heidelberg University Hospital, 69120 Heidelberg, Germany; 8 National Center for Tumor Diseases (NCT) Heidelberg, Department of Translational Medical Oncology, German Cancer Research Center (DKFZ), 69120 Heidelberg, Germany; 9 Department of Neuro-Oncology, Heidelberg University Hospital, 69120 Heidelberg, Germany; wolfgang.wick@med.uni-heidelberg.de

**Keywords:** glioblastoma, radiochemotherapy, microRNA, miR-221, miR-222, miR-17-92, prognosis

## Abstract

MicroRNAs (miRs) are non-coding master regulators of transcriptome that could act as tumor suppressors (TSs) or oncogenes (oncomiRs). We aimed to systematically investigate the relevance of miRs as prognostic biomarkers in primary glioblastoma multiforme (GBM) treated with postoperative radio(chemo)therapy (PORT). For hypothesis generation, tumor miR expression by Agilent 8x15K human microRNA microarrays and survival data from 482 GBM patients of The Cancer Genome Atlas (TCGA cohort) were analyzed using Cox-PH models. Expression of candidate miRs with prognostic relevance (miR-221/222; miR-17-5p, miR-18a, miR-19b) was validated by qRT-PCR using Taqman technology on an independent validation cohort of GBM patients (*n* = 109) treated at Heidelberg University Hospital (HD cohort). In TCGA, 50 miRs showed significant association with survival. Among the top ranked prognostic miRs were members of the two miR families miR-221/222 and miR-17-92. Loss of miR-221/222 was correlated with improved prognosis in both cohorts (TCGA, HD) and was an independent prognostic marker in a multivariate analysis considering demographic characteristics (age, sex, Karnofsky performance index (KPI)), molecular markers (O-6-methylguanine-DNA methyltransferase (MGMT) methylation, IDH mutation status) and PORT as co-variables. The prognostic value of miR-17-92 family members was ambiguous and in part contradictory by direct comparison of the two cohorts, thus warranting further validation in larger prospective trials.

## 1. Introduction

Glioblastoma multiforme (GBM) is the most frequent and aggressive form of primary brain tumors in adults, with an annual incidence of 3.2 per 100,000 [1,2,3]. Despite multimodal therapies, prognosis remains poor, with median overall survival (OS) of 14.5 months [4]. The standard therapy regimen consists of maximal tumor resection followed by postoperative radiotherapy (RT) [5,6] and temozolomide (TMZ)-based chemotherapy [7,8]. The therapy schema might be adapted in elderly patients [6,9,10,11,12]. On a molecular level, O-6-methylguanine-DNA methyltransferase (MGMT) hypermethylation (increased efficacy of alkylating agents [13]) and IDH1/2 mutation (secondary GBM [8,14,15]) are the most important prognostic markers used in clinical routine [16,17,18]. Further molecular mechanisms underlying differences in therapy outcome as well as interindividual heterogeneity in response to radiochemotherapy are not yet well understood, despite efforts to classify tumors based on epigenetic alterations [19,20,21], and require further research aiming for a patient-tailored therapy adjustment. 

MicroRNAs (miRs) are small non-coding RNA molecules that consist of about 22 nucleotides that are able to repress gene expression by posttranscriptional decay of target mRNA. The miR molecule is incorporated into the RNA-induced silencing complex (RISC), which then targets the 3’-untranslated region of its target mRNA. Depending on the grade of complementarity between the miR seed sequence and the mRNA, it is either degraded or its translation is repressed. Missing the necessity for perfect complementarity between the miR and its target mRNA allows for the regulation of one target gene through several miRs as well as the regulation of several genes by one single miR [22]. The involvement of miRs in different biological processes, such as cell survival, apoptosis, migration, invasion, switch of dormant tumor cells to fast-growth, and response to RT, has been shown, and abnormal expression patterns were observed in GBM [23,24,25,26]. Thus, differential expression of single miRs or signatures of miRs might be linked to enhanced tumorigenicity and influence patients’ outcome [23,27]. 

The Cancer Genome Atlas’ (TCGA) primary GBM cohort currently presents the largest repository of integrative molecular and clinical data for GBM and is frequently used for the hypothesis generation and validation of newly discovered biomarker [28]. However, validation cohorts with more comprehensive clinical information are required to confirm the relevance of discovered biomarkers by large-scale data. Therefore, we aimed to examine the prognostic value of miR expression with OS in GBM, using the TCGA cohort as the discovery dataset and a cohort with primary GBM patients treated at Heidelberg University Hospital for validation of the selected miRs. 

## 2. Results

### 2.1. Patient Characteristics

Patient characteristics are shown in Table 1. The evaluated TCGA cohort contained miR expression data from 482 patients; 109 patients were included in the University Hospital (HD) cohort. Median survival was 14.0 (TCGA) and 15.2 months (HD), with median follow-up times of 55.7 and 66.7 months, respectively. Median age at diagnosis was 59 years (TCGA) and 65 years (HD). Higher fractions of males were included (61% and 65% for TCGA and HD, respectively). Karnofsky performance index (KPI) greater or equal to 80% was reported in 58% (TCGA) and 39% (HD), with KPI below 80% reported in 19% (TCGA) and 24% (HD). MGMT promoter hypermethylation was observed in 10% (TCGA) and 39% (HD); hypomethylation was observed in 28% (TCGA) and 35% (HD). For 62% (TCGA) and 26% (HD), data was missing. Absence of IDH1 mutation was proven for 97% for the HD data and for 68% for the TCGA data set, while an additional 26% remained unknown and only 6% showed an IDH1 mutation. In HD, all patients with IDH1 mutated tumors had been excluded from the study, still the IDH1 mutation status remained unknown for 3%. Patients were treated with temozolomide (TMZ) (TCGA: 58%, HD: 80%), and 64% (TCGA) and 87% (HD) of the patients received standard PORT with radiation dosage ≥ 40 Gy (missing information: 33% TCGA, 7% HD) 

**Figure 1:**
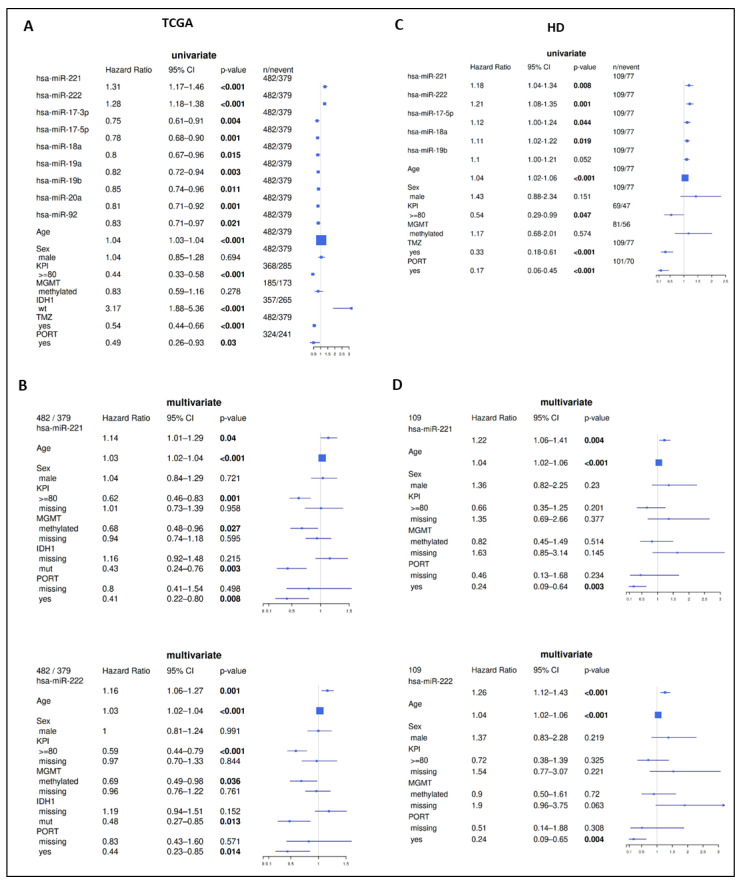
Uni- and multivariate analyses of miR expression for OS. Univariate analyses show a negative prognostic value for miR-221/222 ((**A**) TCGA and (**C**) HD). Multivariate analyses confirm their prognostic value (**B**,**D**). For members of the miR-17-92 cluster, a positive prognostic value was observed in the TCGA cohort (**A**); in HD, an unfavorable prognosis was found (**C**). Bold font indicates *p*-value ≤ 0.05 (Cox proportional hazard models, HR: hazard ratio; CI: confidence interval).

In both the TCGA and HD cohort, clinical covariates age and KPI as well as treatment with TMZ and PORT showed significant association with OS (Figure 1). IDH1 mutation was associated with better prognosis in the TCGA cohort. 

Treatment and molecular covariates showed expected behavior with regard to outcome. Missing data, especially in the TCGA cohort, however, increases uncertainty in the interpretation of the findings.

### 2.2. Prognostic Value of miRs in TCGA

**Figure 2:**
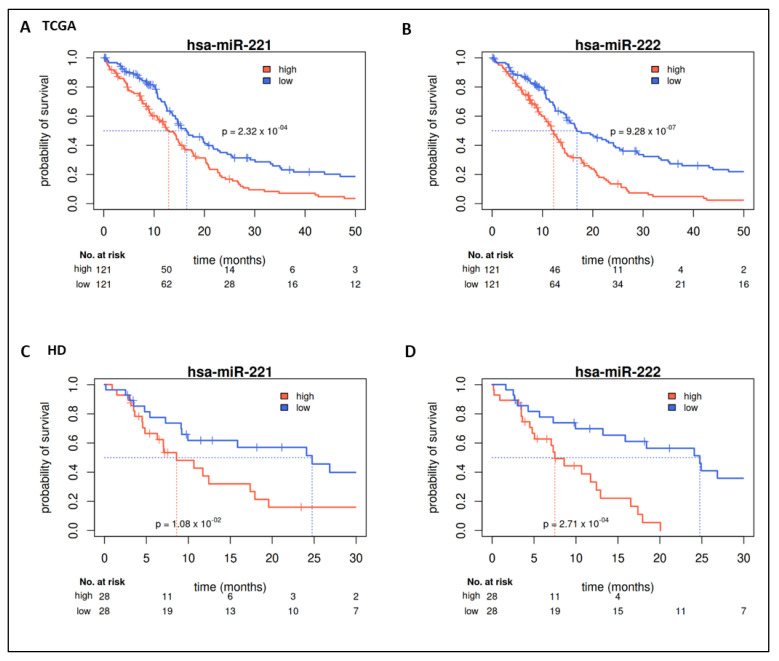
Prognostic value of miR-221 and miR-222. Kaplan–Meier survival curves for overall survival. High expression of miR-221 ((**A**): TCGA; (**C**): HD) and miR-222 ((**B**): TCGA, (**D**): HD) is associated with shorter OS in both cohorts. Median survival differences are 3.6 (16, HD cohort) months for miR-221 and 4.7 (17, HD cohort) months for miR-222 (Kaplan–Meier estimators and likelihood ratio test for groups of patients with expression below the first vs. above the third quartile).

**Figure 3:**
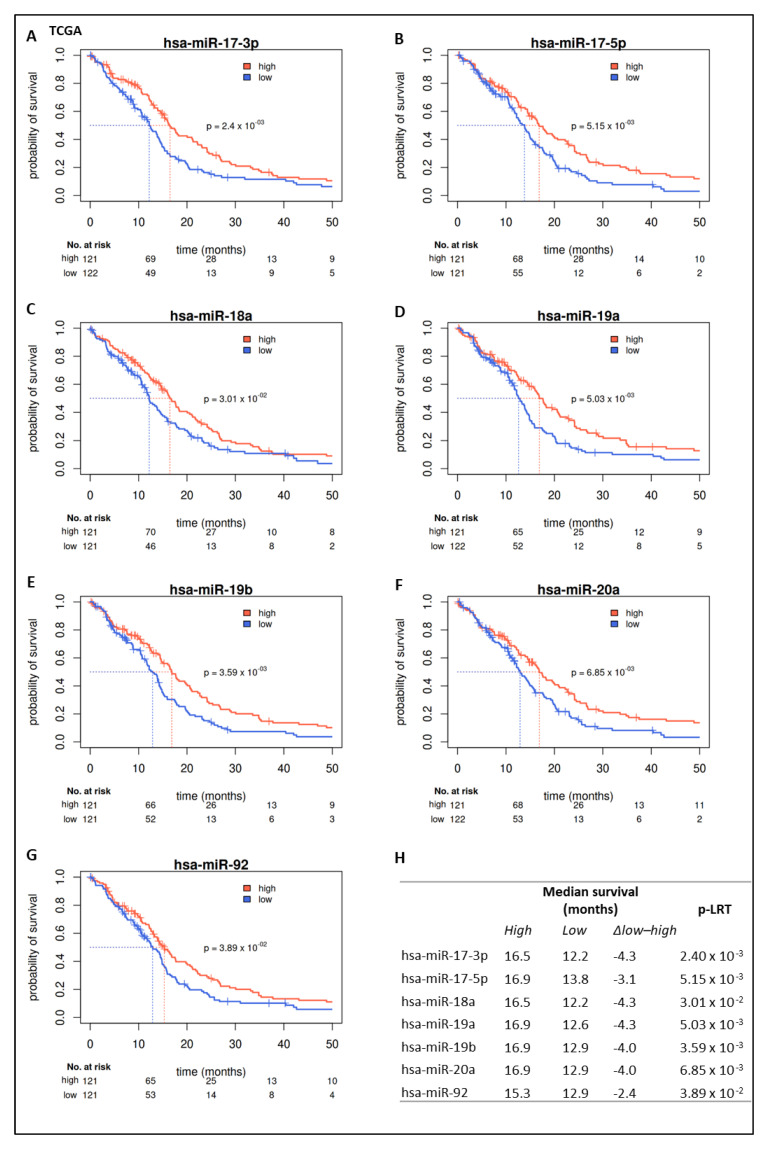
Prognostic value of miR-17-92 family in the TCGA cohort. Kaplan–Meier survival curves for overall survival. High expression of miR-17-3p (**A**), miR-17-5p (**B**), miR-18a (**C**), miR-19a (**D**), miR-19b (**E**), miR-20a (**F**), and miR-92 (**G**) is associated with shorter OS. Median survival differences and *p*-values are shown in (**H**) (Kaplan–Meier estimators and likelihood ratio tests for groups of patients with expression below the first vs. above the third quartile).

We evaluated miR expression in the TCGA-GBM cohort (above upper vs. below lower quartile expression defined groups) with regard to their prognostic value. For a total of 50 miRs, a significant survival difference (*p*-value < 0.05) could be detected (Supplementary Table S1). The first three miRs (hsa-miR-222, hsa-miR-148a, hsa-miR-221) showed significant differences with a false discovery rate (FDR) < 0.05. The 20 highest ranked miRs (by *p*-value) are shown in Table 2. Seven out of these miRs belong to two miR clusters, namely miR-221/222 and miR-17-92. Corresponding median survival differences are shown in Table 2. High expression of miR-221/222 was associated with shorter survival (Figure 2A,B, Table 2); high expression of members of the miR-17-92 cluster, however, was associated with prolonged survival (Figure 3, Table 2). 

Univariate evaluation of continuous expression of miRs confirmed these findings (Figure 1A). Multivariate analysis confirmed the value of miR-221/222 as an independent prognostic factor in GBM (Figure 1B). In contrast, multivariate analyses did not yield significant results for the members of the miR-17-92 cluster (Supplementary Figure S1).

### 2.3. Validation in HD Cohort

MiR-221/222 and miR-17-5p/-18a/-19b were quantified by qRT-PCR in the HD cohort. Their prognostic value was evaluated utilizing the same approach applied to the TCGA cohort (i.e., upper vs. lower quartile). In line with the training cohort, patients of the HD cohort with tumors expressing high levels of miR-221 or miR-222 showed a significantly decreased OS compared to patients expressing low levels of miR-221 or miR-222 (Figure 2C,D). Median survival difference between groups with high and low expression was 16 months for miR-221 and 17 months for miR-222. In addition, continuous expression of miR-221 (HR 1.18 [1.04–1.34]) and miR-222 (HR 1.21 [1.08–1.35]) could be confirmed as prognostic markers for OS in univariate analyses (Figure 1C). Multivariate analyses considering demographic characteristics (age, sex, KPI), molecular markers (MGMT methylation, IDH mutation status), and PORT as covariables validated the independency of the prognostic value of miR-221 (HR 1.22 [1.06–1.41]) and miR-222 (HR 1.26 [1.12–1.43]). Only age and PORT were significant prognosticators (Figure 1D). 

**Figure 4:**
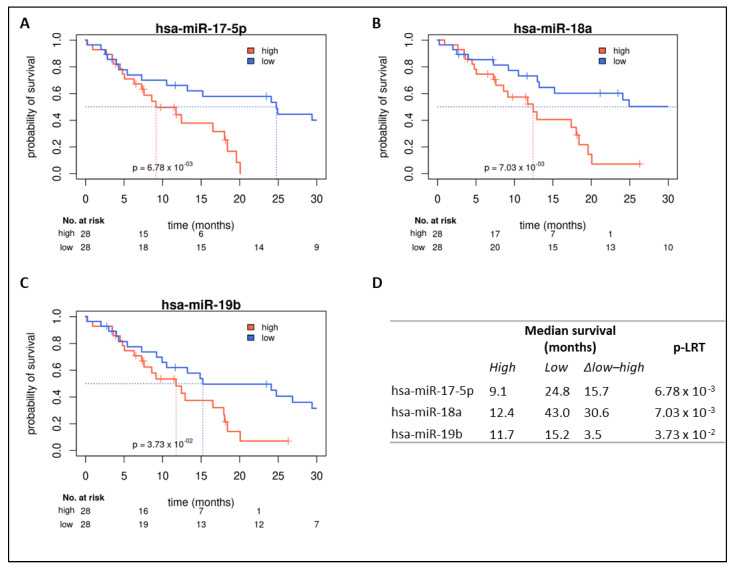
Prognostic impact of members of miR-17-92 in the HD cohort. Kaplan–Meier survival curves for overall survival. High expression of miR-17-5p, miR-18a, and miR-19b is associated with shorter overall survival (**A**–**C**), with median survival differences of 3.5 to 30.6 months. Median survival differences and *p*-values are shown in (**D**) (Kaplan–Meier estimators and likelihood ratio tests for groups of patients with expression below the first vs. above the third quartile).

For all investigated members of the miR-17-92 cluster (miR-17-5p, miR-18a, miR-19b), a significant prognostic separation could be observed showing unfavorable prognosis in the high-expressing group (Figure 4). This contradicts findings from the TCGA cohort (favorable prognosis in the miR-17-92 cluster high-expressing group, Figure 3). In parallel, univariate survival analysis of continuous miR expression showed a significant unfavorable prognostic value for miR-17-5p (HR 1.12 [1.00–1.24]) and miR-18a (HR 1.11 [1.02–1.22]) (Figure 1C). Multivariate analyses considering demographic characteristics, molecular markers, and PORT as co-variables confirmed miR-17-5p, miR-18a, and miR-19b as independent prognostic markers, with only the two co-variables age and PORT being additional significant independent factors (Supplementary Figure S2). 

### 2.4. Prognostic Value in Covariate Stratified Subcohorts

Comparison of patients’ characteristics of the TCGA and the HD cohort revealed a certain degree of cohort heterogeneity, as outlined in Table 1. Therefore, the prognostic value of miRs was assessed in subcohorts, formed depending on clinical characteristics (e.g., age, sex, KPI), known molecular prognostic factors (MGMT and IDH1 status), as well as received therapy (TMZ, PORT). A total of 18 (TCGA) and 16 (HD) subcohorts with group sizes between 16 and 328 patients for TCGA and 6 and 95 patients for the HD cohort were defined for the univariate evaluation of continuous expression of miRs of interest. In the TCGA cohort, univariate analyses confirmed a negative prognostic value with significant results for miR-221 in 11 out of 20 subgroups and for miR-222 in 13 out of 20 subgroups (Supplementary Figure S3). In the HD cohort, significant results were achieved for miR-221 in 8 out of 16 subgroups and for miR-222 in 10 out of 16 subgroups, though small group sizes may have prevented significant results from being obtained in more subgroups. Noteworthy, in TCGA as well as in HD, significant results were in particular reached in subcohorts composed of younger patients, patients with high KPI, and patients treated with standard therapy scheme (PORT, TMZ), whereas miR expression had no prognostic influence in IDH1-mutated GBM (TCGA). 

For members of the miR-17-92 cluster, the subgroup analyses in TCGA and HD yielded more heterogeneous results. Significant results were achieved in only 4 to 8 out of 18 (TCGA) and 3 to 5 out of 16 (HD) subgroups (Supplementary Figure S4). However, a general trend towards a positive (TCGA) and a negative prognostic value (HD) could be observed (Supplementary Figure S4). 

Thus, covariates putatively modulating the effect of miRs could not be identified in either multivariate analyses or in these subcohort analyses. 

## 3. Discussion

**Figure 5:**
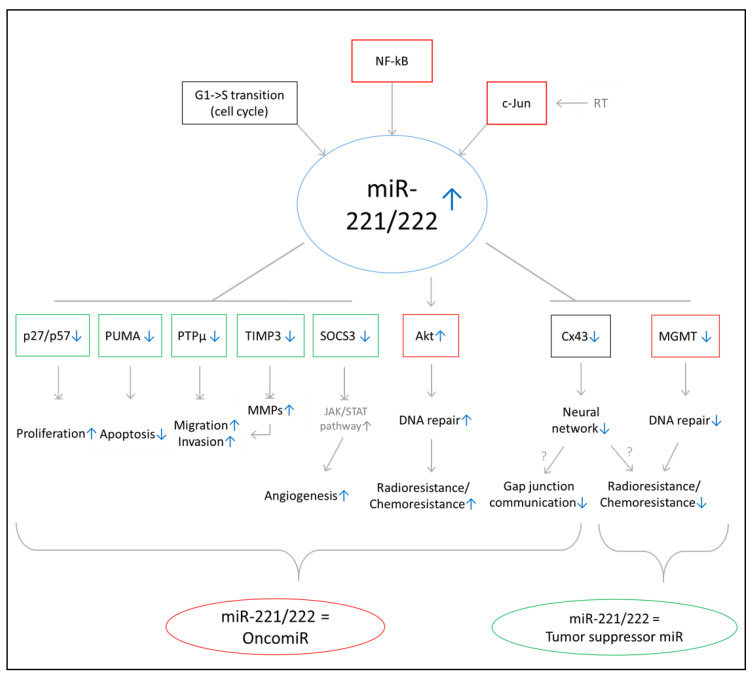
Summary of validated target genes and postulated cellular functions of miR-221/222. Most previous reports have proposed a pro-tumorigenic function of miR-221/222, whereas only a few studies have postulated a tumor-suppressive activity. Candidate target genes of this family and their reported involvement in cellular processes are shown. Green colored framing indicates potential tumor suppressive function of corresponding gene or miR. Red colored framing indicates potential pro tumorigenic function of corresponding gene or miR. Blue arrows indicate direction of regulation for corresponding pathway, miR and gene regulation.

We report the expression of miR-221 and miR-222 as important prognostic factors in both the TCGA training (*n* = 482) and HD validation cohort (*n* = 109). MiR-221 and miR-222 are clustered together on chromosome Xp11.3 and have similar target specificity [29]. Their expression has been attributed to GBM [30,31,32,33,34,35]. The majority of previously published functional in vitro experiments suggests a pro-tumorigenic activity for the miR-221/222 family (Figure 5). Confirmed target genes underline their involvement in promoting cell cycle progression and proliferation (p27, p57), migration and invasion (PTPµ and TIMP3), DNA repair (Akt), radio- and chemo resistance (Akt), and angiogenesis (SOCS3) as well as inhibiting apoptosis (PUMA) and gap junction communication (Cx43) [34,36,37,38,39,40]. However, negative regulation of some target genes, such as MGMT and Cx43, may also elicit contrary effects and sensitize GBM to therapy (e.g., via inhibition of DNA repair (MGMT), inhibition of microtubule-dependent proliferation and invasion (Cx43), and enhancing radio- and chemo resistance (MGMT, Cx43)) [41,42,43,44]. Therefore, mechanistic studies are needed to explore the net effect of miR-221/222 expression on tumor microenvironment communication and tumor response to therapy. So far, miR-221/222 knockdown has been shown to reduce xenograft tumor growth in mouse models in vivo [30,35,39,40]. Overall, the majority of these studies point to an oncogenic function of miR-221/222, in alignment with our observation that reduced miR-221/222 expression correlated with improved OS in both studied cohorts of primary GBM patients.

Table 3 summarizes the previously published data of survival analyses of GBM patients for miR-221/222. For the TCGA cohort, a correlation between miR-221/222 expression and OS had previously been reported, demonstrating a negative prognostic impact; however, Delfino et al. showed a positive prognostic correlation of miR-221 in a subgroup of patients who received RT [45,46,47]. Apart from TCGA, Li et al. quantified the expression level of miR-221/222 in 54 GBM samples by qRT-PCR and confirmed the correlation between high miR-221/222 expression and poor outcome [30]. Similarly, Chen et al. showed a survival benefit for patients with low expression of miR-221 [48]. In three studies (two with GBM, one with glioma patients), the peripheral blood concentration of miR-221/222 showed a negative prognostic impact [49,50,51]. Additionally, a meta-analysis of 1204 patients with different tumor entities, including glioma patients, revealed an association between high expression of miR-221/222 and poor survival [52] (Table 3). Together with the present study, these data lend support to the concept that miR-221/222 may elicit a net pro-tumorigenic effect as “onco-miRs” in GBM pathology. Functional perturbation of miR-221/222 may constitute a novel and attractive venue for targeted intervention in GBM patients that warrants further studies. 

**Figure 6:**
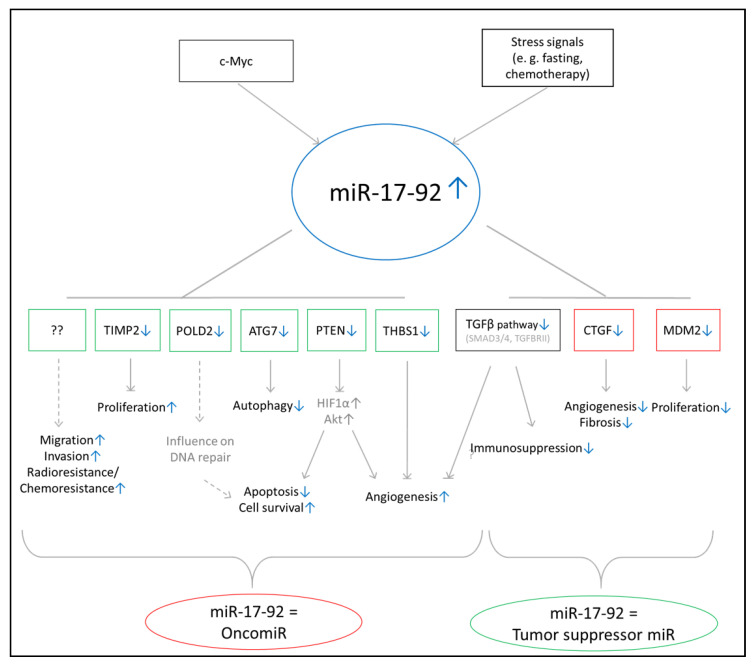
Summary of validated target genes and postulated cellular functions of miR-17-92. Contradictory mechanisms of action (MoAs) for this family have been proposed in previous literature (i.e., both pro-tumorigenic and tumor suppressive activities have been reported for members of the miR-17-92 cluster). Candidate target genes and their reported involvement in cellular processes are shown. Green colored framing indicates potential tumor suppressive function of corresponding gene or miR. Red colored framing indicates potenitial pro tumorigenic function of corresponding gene or miR. Blue arrows indicate direction of regulation for the corresponding pathway, miR and gene.

The miR-17-92 cluster consists of miR-17-3p, miR-17-5p, miR-18a, miR-19a, miR-19b, miR-20a, and miR-92a and was found to be upregulated in GBM cell lines and patient tumor samples [53,54,55]. Both the pro-tumorigenic as well as tumor-suppressive activity of the miR-17-92 cluster in GBM has previously been reported. The miR-17-92 cluster seems to be activated by c-Myc and cellular stress signals [56,57]. Confirmed target genes implicating an oncomiR function suggest their involvement in promoting proliferation (TIMP2), DNA repair and cell survival (POLD2, PTEN), and angiogenesis (TGFβ pathway, THBS1, PTEN) and inhibiting autophagy (ATG7) and apoptosis (POLD2) [53,55,56,57,58]. Additional in vitro experiments have showed the promotion of migration, invasion, and radio- and chemo resistance [58,59,60]. On the contrary, a tumor-suppressive function is proposed by the inhibition of proliferation (via MDM2), immunosuppression (via TGFβ), as well as angiogenesis and fibrosis (via CTGF) [57,61] (Figure 6).

Additionally, in vitro experiments in cell lines other than GBM investigating the oncogenic properties of miR-17-92 showed the increased aggressiveness of different types of tumor cells when overexpressing members of the miR-17-92 cluster (e.g., overexpression in lymphoma cells led to increased cell growth and cell cycle progression and decreased apoptosis) [62,63,64]. Correspondingly, the overexpression of miR-17-92 family in a B-cell lymphoma and a neuroblastoma in vivo mouse model led to increased tumorigenesis [65,66]. 

Our survival analyses of members of the miR-17-92 cluster yielded contradicting results: within the TCGA cohort, high expression was associated with longer OS in univariate and multivariate analyses. In contrast, the HD cohort showed shorter survival for patients with tumors expressing high levels of miR-17-92 members. In Table 4, previous publications of survival analyses of miR-17-92 members in GBM patients are summarized. Based on data from the TCGA cohort, Fox et al. proposed that high expression of miR-18a together with low expression of a TGFβ gene signature correlated with improved outcome [61]. Yuan et al. showed that high expression of a four-miR signature including miR-17-5p was associated with longer OS [67]. On the contrary, a study by Zhao et al. showed a negative prognostic impact of high expression of miR-17-5p and miR-20a [51]. Correlation of miR-17-92 expression with OS in other tumor entities such as colorectal cancer, neuroblastoma, lung cancer, multiple myeloma, and esophageal squamous cell carcinoma showed poorer outcomes for patients with high expression levels [68,69,70,71,72,73]. 

Studies exploring the effect of miRs tend to be more heterogenous and may even produce contradictory results, which might arise from a number of issues, including the following: small patient cohorts with high uncertainty of clinical data from pathological annotation to treatment homogeneity; technique immanent problems in microarray-based screens (normalization, transformation, multiplicity adjustment in screening testing), which are further complicated by the short sequence with high sequence homology across a miR family and most importantly the often low abundance of miRs, leading to high CT values in PCR-based methods and close to background microarray intensities. These factors might limit the reproducibility of hypotheses generated in the TCGA cohort, as found for the miR-17-92 in our study. Therefore, reliable analyses require uniformly treated patients, valid methods for verification of screening results, and patient groups of sufficient size. 

Additionally, further mechanistic studies investigating the cellular functions of miR-221/222 and miR-17-92 in GBM are needed. Interestingly, both of the investigated miR families are reported to increase GBM cell invasiveness, while infiltrative growth is characteristic for GBM cells and was postulated to be linked to enhanced radio-resistance of GBM cells [74]. While high-LET (linear energy transfer) particle irradiation with, for example, carbon ions has been shown to eradicate radio-resistant GBM cells, it would be interesting to see if high-LET irradiation would also overcome the pro-invasive and radioresistant effect of miR-221/222 and miR-17-92 [75,76,77]. 

Taken together, miR-221/222 are promising prognosticators of OS in primary GBM, and could be confirmed as prognostic markers in two independent cohorts, namely the TCGA (*n* = 482) and the HD (*n* = 109) cohort. Ambiguous results regarding the expression of members of the miR-17-92 family require further exploration. 

## 4. Materials and Methods 

### 4.1. TCGA GBM Collective 

For training, level 3 expression data generated on Agilent 8x15K human microRNA microarrays by TCGA GBM Network were downloaded and utilized [28]. Clinical data of TCGA GBM patients were manually curated and supplemented with information on IDH1 mutation status by analysis of TCGA level 3 sequencing data. Level 3 whole-exome sequencing data was retrieved through the GDC data portal. Samples showing any non-silent IDH1 mutation in any of the four variant calling pipeline data were considered IDH1 mutant. The complete data set included the miR expression data of 534 samples corresponding to 490 patients; for eight patients, no survival information was available. These samples were excluded. For five patients, two samples were available—their median value was used for further analysis. Due to missing control tissue, miR expression data was virtual pool normalized by dividing each value by the median for this miR of all patients.

### 4.2. Heidelberg GBM Collective

#### 4.2.1. Patients and Tumor Samples

One hundred and nine patients with primary GBM were included in this study, all of whom received surgery at the Department of Neurosurgery, University of Heidelberg. Clinical data were obtained from patient charts. 

#### 4.2.2. Ethics Approval and Consent to Participate

All patient consented to participate in this study, and ethical approval was obtained by the IRB-Ethics Committee of the Medical Faculty of Heidelberg University (approval number 005/2003).

#### 4.2.3. Patient Material

Tumor samples were acquired through biopsy/surgical resection. Samples were assessed by board-certified neuropathologists for diagnosis of GBM and tumor fraction of at least 60%. IDH1 mutation and MGMT promotor methylation status were determined as previously described [16,78,79]. 

#### 4.2.4. Nucleic Acid Isolation

RNA extraction from surgically acquired specimens was performed using TRIzol^®^ RNA Isolation Reagent (#15596, Life Technologies, Carlsbad, CA, USA) or AllPrep^®^ DNA/RNA/Protein Mini Kits (#80004, Qiagen, Hilden, Germany) in combination with RNeasy^®^ MinElute^®^ Cleanup Kit (#74204, Qiagen). RNA concentrations were measured with the NanoDrop 1000 Spectrometer (Thermo Fisher Scientific, Waltham, MA, USA), and RNA quality control was performed with the 2100 Bioanalyzer system (Agilent, Santa Clara, CA, USA). All steps were performed according to the manufacturers’ protocols. Total RNA from TRIzol^®^-processed samples and supernatant from AllPrep^®^ purified samples were used for further analyses. 

#### 4.2.5. microRNA Analysis

Expression of miRs was quantified by real-time PCR on an ABI 7900 system (Life Technologies). For reverse transcription, TaqMan^®^ MicroRNA Reverse Transcription Kit (#4366597, Thermo Fisher Scientific) was used, and for quantitative real-time PCR TaqMan^®^ Universal PCR Master Mix II (no UNG) (#4440048, Thermo Fisher Scientific) was used. Primers were ordered at TaqMan^®^ (Thermo Fisher Scientific): hsa-miR-221 (#000524), hsa-miR-222 (#002276), hsa-miR-17-5p (#00393), hsa-miR-18a (#002422), hsa-miR-19b (#000396), controls RNU44 (#001094), and U6_snRNA (#001973). Measured CT values were normalized against the arithmetic mean of controls (RNU44 and U6_snRNA). Data were further virtual pool normalized to the arithmetic mean of each miR. 

### 4.3. Statistical Analyses

Statistical analyses were conducted in R v. 3.6.2 [80]. Survival analyses were performed with the survival package [81]. Cox proportional hazard models (Cox-PH) were used for analyses, likelihood ratio tests are reported if not indicated otherwise. Kaplan–Meier estimators were used to calculate median overall survival; median follow-up times were obtained with the inverse Kaplan–Meier method. Plots were created with the dataAnalysisMisc package [82]. For identification of miRs associated with prognosis, upper and lower quartile expression-derived groups (per miR) were compared in the TCGA cohort. Multiplicity adjustment (FDR) was performed using the Benjamini–Hochberg method. Significance level alpha was set to 0.05 (two-sided). 

## Figures and Tables

**Table 1:** Patients’ characteristics of The Cancer Genome Atlas (TCGA) and Heidelberg University Hospital (HD) cohort. OS: overall survival; FU: follow-up period; KPI: Karnofsky performance index; MGMT: O-6-methylguanine-DNA methyltransferase; TMZ: temozolomide; PORT: postoperative radiotherapy.

		TCGA		HD	
Feature		*N*	%	*N*	%
All		482	100	109	100
Median OS (months)	(95% CI)	14.0 (12.7–15.0)		15.2 (10.7–24.1)	
Median FU (months)		55.7		66.7	
Age (years)	median (range)	59 (0–89)		65 (20–86)	
Age category I					
	<60 years	249	51.7	43	39.4
	≥60 years	233	48.3	66	60.6
Age category II					
	<50 years	120	24.9	20	18.3
	50–59 years	129	26.8	23	21.1
	60–69 years	134	27.8	38	34.9
	≥70 years	99	20.5	28	25.7
Sex					
	female	186	38.6	38	34.9
	male	296	61.4	71	65.0
KPI					
	<80	89	18.5	26	23.9
	≥80	279	57.9	43	39.4
	N/A	114	23.7	40	36.7
MGMT promotor					
	hypo-methylated	135	28.0	38	34.9
	methylated	50	10.4	43	39.4
	N/A	297	61.6	28	25.7
IDH1					
	mutation	29	6.0	0	0
	wildtype	328	68.0	106	97.2
	N/A	125	25.9	3	2.8
TMZ					
	no	202	41.9	22	20.2
	yes	280	58.1	87	79.8
PORT					
	yes	308	63.9	95	87.2
	no	16	3.3	6	5.5
	N/A	158	32.8	8	7.3

**Table 2:** The 20 top-ranked miRs from the TCGA glioblastoma multiforme (GBM) cohort associated with prognosis.

	Median Survival (Months)			
	High	Low	ΔLow–High	p-LRT
**hsa-miR-222**	12.2	16.9	4.7	9.28 × 10^−7^
hsa-miR-148a	12.6	17.5	5.1	5.05 × 10^−5^
**hsa-miR-221**	12.9	16.5	3.6	2.32 × 10^−4^
hsa-miR-200a	13.7	15.9	2.2	9.01 × 10^−4^
hsa-miR-106a	16.9	12.5	−4.4	1.28 × 10^−3^
hsa-miR-212	13.6	15.9	2.3	1.50 × 10^−3^
hsa-miR-200b	12.4	16.8	4.4	2.40 × 10^−3^
**hsa-miR-17-3p**	16.5	12.2	−4.3	2.40 × 10^−3^
hsa-miR-183	15.9	11.3	−4.6	2.47 × 10^−3^
hsa-miR-140	15.6	12.2	−3.4	2.89 × 10^−3^
hsa-miR-340	16.0	13.8	−2.2	3.18 × 10^−3^
hsa-miR-21	12.6	14.8	2.2	3.20 × 10^−3^
hsa-miR-34b	14.2	16.5	2.3	3.57 × 10^−3^
**hsa-miR-19b**	16.9	12.9	−4.0	3.59 × 10^−3^
hsa-miR-34a	14.4	15.9	1.5	3.99 × 10^−3^
**hsa-miR-19a**	16.9	12.6	−4.3	5.03 × 10^−3^
**hsa-miR-17-5p**	16.9	13.8	−3.1	5.15 × 10^−3^
**hsa-miR-20a**	16.9	12.9	−4.0	6.85 × 10^−3^
hsa-miR-487a	13.3	15.0	1.7	9.81 × 10^−3^
hsa-miR-382	11.5	15.0	3.5	1.11 × 10^−2^

(Likelihood ratio test (LRT) for groups of patients with expression below the first vs. above the third quartile; *n* = 482 patients; bold font indicates members of the miR-221/222 and miR-17-92 family).

**Table 3:** Summary of previously published data on the correlation between miR-221/222 expression and survival of GBM patients.

	Number of Samples (Method, Tissue)	miR-221 HR (95% CI)	*p* Value	miR-222 HR (95% CI)	*p* Value
Delfino 2011 [47]	253 (TCGA, microarray)	0.41 (0.22–0.75) *	0.0298	2.14 (1.51–3.03)	<0.0001
Wang 2014 [52]	1204 (meta-analysis) ^+^	1.91 (1.28–2.85)	0.002	2.15 (1.51–3.06)	<0.0001
Zhang 2015 [49]	50 (qRT-PCR, plasma samples)	2.40 (1.42–4.05)	N/A ^#^	2.81 (1.70–4.65)	N/A ^#^
Li 2016 [30]	54 (qRT-PCR)	2.18 (1.02–4.65)	0.044	2.13 (1.01–4.48)	0.043
Yerukala 2016 [46]	247 (TCGA, microarray)	MED = 0.129 ^&^		MED = 0.797 ^&^	
Zhao 2017 [51]	106 (microarray, serum samples)	N/A		1.71 (1.07–3.63) ^%^	0.038
Chen 2018 [48]	114 (qRT-PCR)	N/A Survival benefit when miR ↓	0.027 ^§^	N/A	0.796 ^§^
Swellam 2019 [50]	20 (qRT-PCR, blood samples)	N/A Survival benefit when miR ↓	0.002	N/A Survival benefit when miR ↓	0.001
Sun 2019 [45]	458 (TCGA, microarray)	N/A		1.28 (1.18–1.38)	<0.001
TCGA	482 (TCGA, microarray)	1.31 (1.17–1.46)	<0.001	1.28 (1.18–1.38)	<0.001
HD	109 (HD, qRT-PCR)	1.18 (1.04–1.34)	0.008	1.21 (1.08–1.35)	0.001

(* Subgroup of patients treated only with RT; ^+^ includes other tumor entities; ^#^ no *p* value available; ^&^ MED: main effect difference; ^%^ 2-year disease-free survival, ^§^ only MGMT unmethylated GBM; ↓ low expression of miR HR: hazard ratio; CI: confidence interval).

**Table 4:** Summary of previously published data on the correlation between miR-17-92 expression and the survival of GBM patients.

	Number of Samples (Method, Tissue)	Correlation of		HR	*p* Value
		miR Expression	Survival		
Fox 2013 [61]	N/A (TCGA, microarray)	miR-18a↑ (+TGFβ signature↓)	↑	N/A	0.035
Yuan 2017 [67]	48 (qRT-PCR)	miR signature incl. miR-17-5p↑ *	↑ *	N/A	0.0012 *
Zhao 2017 [51]	106 (microarray, serum samples)	miR-17-5p↑	↓	1.7 (1.05–4.01) ^%^	0.043%
		miR-20a↑	↓	1.69 (1.06–3.79) ^%^	0.04%
TCGA	482 (microarray)	miR-17-3p↑	↑	0.75 (0.61–0.91)	0.004
		miR-17-5p↑	↑	0.78 (0.68–0.9)	0.001
		miR-18a↑	↑	0.8 (0.67–0.96)	0.015
		miR-19a↑	↑	0.82 (0.72–0.94)	0.003
		miR-19b↑	↑	0.85 (0.74–0.96)	0.011
		miR-20a↑	↑	0.81 (0.71–0.92)	0.001
		miR-92↑	↑	0.83 (0.71–0.97)	0.021
HD	109 (qRT-PCR)	miR-17-5p↑	↓	1.12 (1.00–1.24)	0.044
		miR-18a↑	↓	1.11 (1.02–1.22)	0.019
		miR-19b↑	↓	1.1 (1.00–1.21)	0.052

(* miR signature consisting of let-7g-5p, miR-139-5p, miR-17-5p, and miR-9-3p; ^%^ 2-year disease-free survival; ↓: low expression of miR; ↑: high expression of miR. HR: hazard ratio; CI: confidence interval).

## Data Availability

Publicly available datasets were analyzed in this study. This data can be found here: https://portal.gdc.cancer.gov/ (accessed on 16 December 2019). Available from the authors upon reasonable request.

## Supplementary Materials

Supplementary Materials can be found at https://www.mdpi.com/1422-0067/22/6/2960/s1. Figure S1: Multivariate analyses of miR expression and clinical features for members of the miR-17-92 family for OS in the TCGA cohort, Figure S2:Multivariate analyses of miR expression and clinical features for members of the miR-17-92 family for OS in the HD cohort, Figure S3:Univariate analysis of miR-221/222 expression for OS in clinically relevant subgroups of the HD and TCGA cohorts, Figure S4: Univariate analysis of miR expression and clinical features in subgroups for miR-17-5p, miR-18a and miR-19b for OS in the TCGA and HD cohort, Table S1: MiR candidates from the TCGA GBM cohort showing significant association between OS and expression levels.

## Abbreviations

GBM	glioblastoma multiforme
HD	Heidelberg
MGMT	O-6-methylguanine-DNA methyltransferase
miR	microRNA
OS	overall survival
RT	radiotherapy
TCGA	The Cancer Genome Atlas
