# Supplementary material for: Prognostic Value of microRNA-221/2 and 17-92 Families in Primary Glioblastoma Patients Treated with Postoperative Radiotherapy
Source: Int J Mol Sci. 2021 Mar 15;22(6):2960. doi: 10.3390/ijms22062960 (PMC7998975; doi:10.3390/ijms22062960)
Supplement: Supplementary file 1 [file ijms-22-02960-s001.pdf]

Suppl. Tab. 1: MiR candidates from the TCGA GBM cohort showing significant association between OS and expression levels (n=482 patients; p <=0.05; Likelihood ratio test (CoxPH models) for groups of patients with expression below the first vs. above the third quartile).

	**Median survival [months]**			
	**high**	**low**	**Δlow-high**	**p-LRT**
hsa-miR-222	12.2	16.85	4.66	9.28e-07
hsa-miR-148a	12.56	17.54	4.98	5.05e-05
hsa-miR-221	12.89	16.49	3.61	2.32e-04
hsa-miR-200a	13.7	15.93	2.23	9.01e-04
hsa-miR-106a	16.85	12.52	-4.33	1.28e-03
hsa-miR-212	13.61	15.93	2.33	1.50e-03
hsa-miR-200b	12.36	16.75	4.39	2.40e-03
hsa-miR-17-3p	16.49	12.2	-4.3	2.40e-03
hsa-miR-183	15.93	11.31	-4.62	2.47e-03
hsa-miR-140	15.61	12.2	-3.41	2.89e-03
hsa-miR-340	16.03	13.84	-2.2	3.18e-03
hsa-miR-21	12.59	14.82	2.23	3.20e-03
hsa-miR-34b	14.16	16.49	2.33	3.57e-03
hsa-miR-19b	16.85	12.89	-3.97	3.59e-03
hsa-miR-34a	14.36	15.9	1.54	3.99e-03
hsa-miR-19a	16.89	12.59	-4.3	5.03e-03
hsa-miR-17-5p	16.85	13.84	-3.02	5.15e-03
hsa-miR-20a	16.89	12.92	-3.97	6.85e-03
hsa-miR-487a	13.25	14.98	1.74	9.81e-03
hsa-miR-382	11.48	14.98	3.51	1.11e-02
hcmv-miR-UL112	15.7	13.61	-2.1	1.27e-02
hsa-miR-9*	15.31	12.92	-2.39	1.52e-02
hsa-miR-505	15.38	13.08	-2.3	1.60e-02
hsa-miR-155	14	14.89	0.89	1.92e-02
hsa-miR-204	12.07	14.66	2.59	1.96e-02
hsa-miR-181c	15.31	12.13	-3.18	1.96e-02

hsa-miR-181d	16.33	13.87	-2.46	2.23e-02
hsa-miR-566	14.66	12.92	-1.74	2.44e-02
hsa-miR-572	14.26	12.13	-2.13	2.45e-02
hsa-miR-488	14.89	12.13	-2.75	2.54e-02
hsa-miR-29c	12.89	14.66	1.77	2.59e-02
hsa-miR-9	16.03	13.93	-2.1	2.84e-02
hsa-miR-18a	16.46	12.2	-4.26	3.01e-02
hsa-miR-106b	15.61	12.2	-3.41	3.08e-02
hsa-miR-801	15.61	13.97	-1.64	3.15e-02
hsa-miR-190	12.92	14.23	1.31	3.15e-02
hsa-miR-324-3p	12.2	14.49	2.3	3.40e-02
hsa-miR-487b	13.93	15.02	1.08	3.41e-02
hsa-miR-490	14.82	13.61	-1.21	3.89e-02
hsa-miR-92	15.31	12.89	-2.43	3.89e-02
hsa-miR-323	12.56	14.69	2.13	3.95e-02
hsa-miR-181b	14.98	13.87	-1.11	4.04e-02
hsa-miR-135b	12.92	14.49	1.57	4.12e-02
hsa-miR-646	14.66	13.97	-0.69	4.15e-02
hsa-miR-409-3p	12.89	15.02	2.13	4.24e-02
hsa-miR-326	14.43	14.49	0.07	4.33e-02
hsa-miR-346	14.82	13.7	-1.11	4.51e-02
hsa-miR-22	13.93	15.61	1.67	4.57e-02
hsa-miR-663	14.66	12.92	-1.74	4.64e-02
hsa-miR-339	14.1	14.85	0.75	4.96e-02

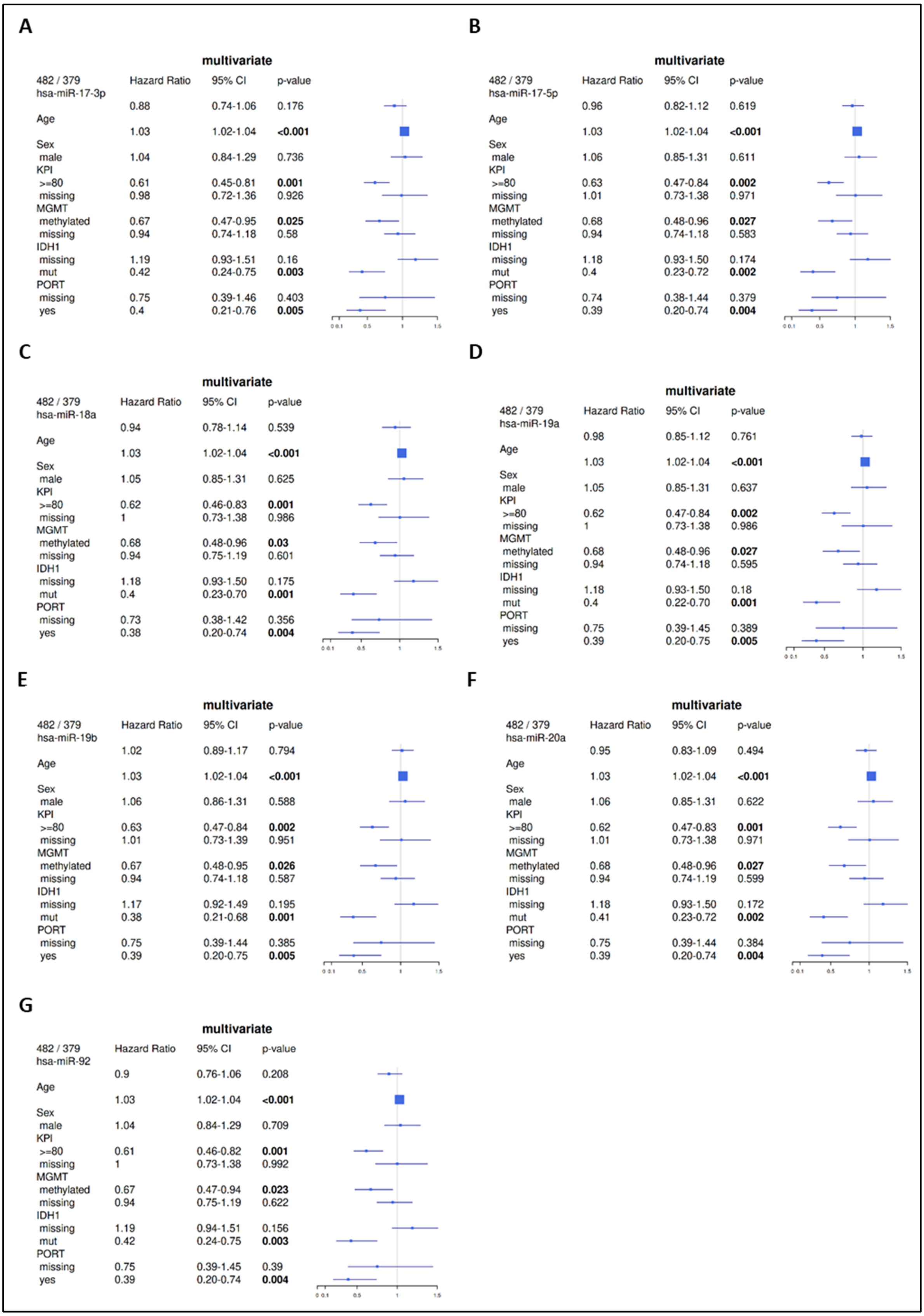

Suppl. Fig. 1: Multivariate analyses of miR expression and clinical features for members of the miR-17-92 family for OS in the TCGA cohort (Cox proportional hazard model, n=482, HR: Hazard Ratio, CI: confidence interval).

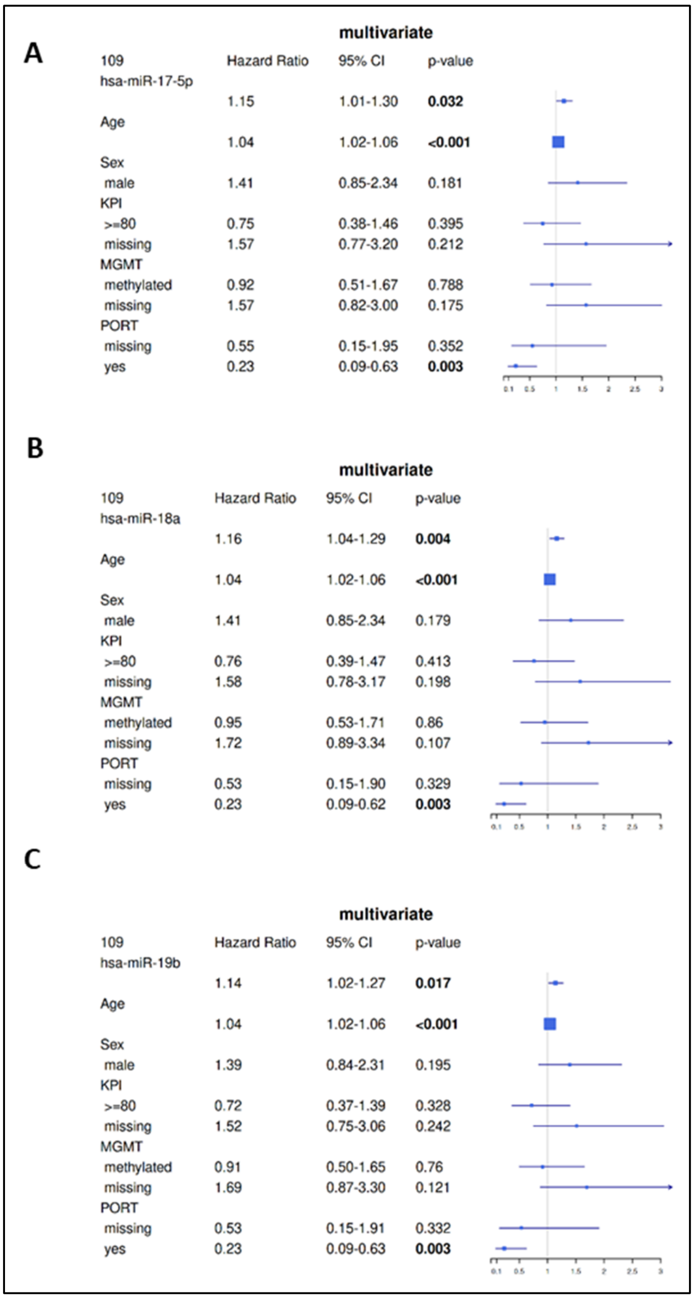

Suppl. Fig. 2: Multivariate analyses of miR expression and clinical features for members of the miR-17-92 family for OS in the HD cohort (Cox proportional hazard model, n=109, HR: Hazard Ratio, CI: confidence interval).

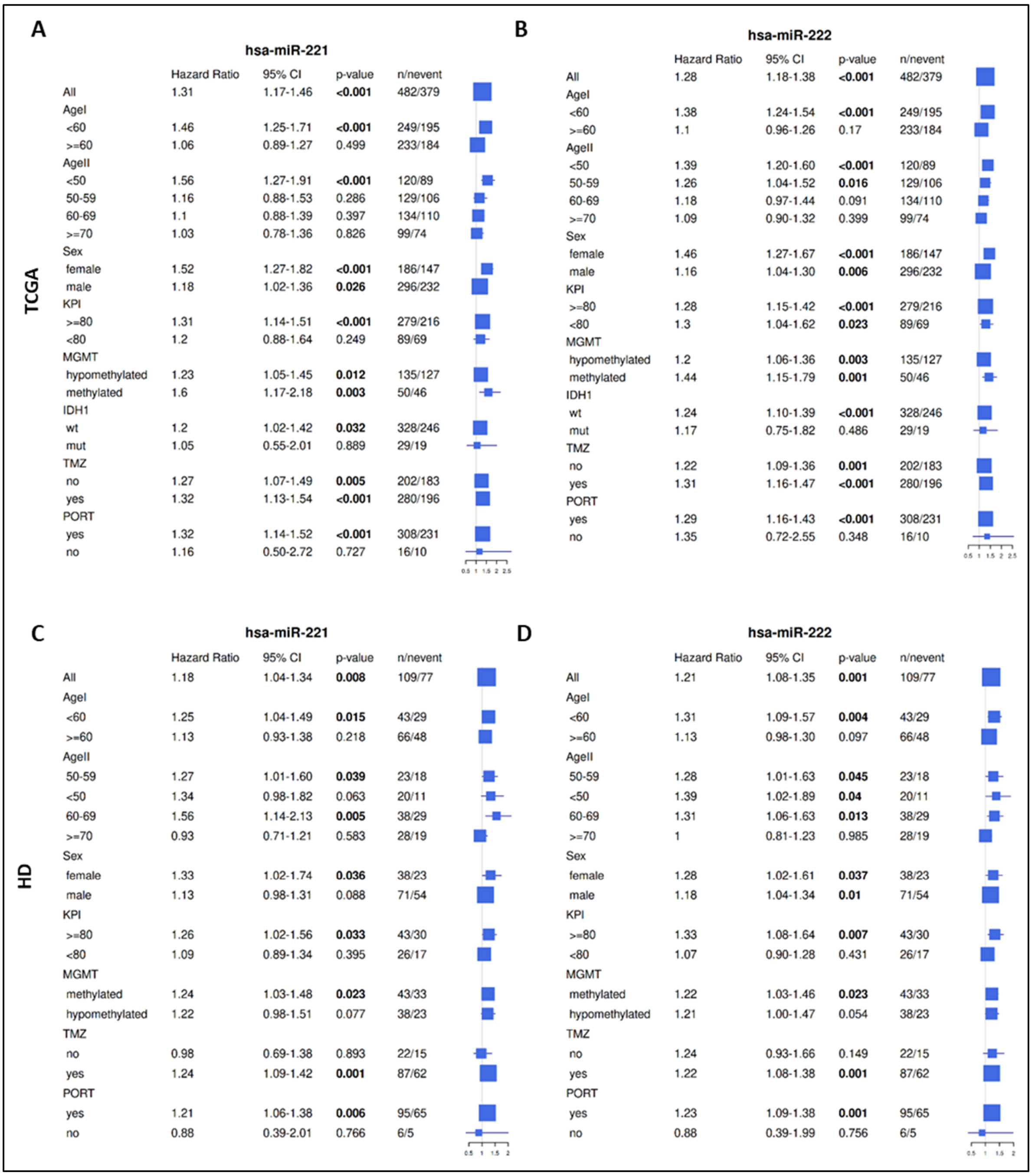

Suppl. Fig. 3: Univariate analysis of miR-221/222 expression for OS in clinically relevant subgroups of the HD and TCGA cohorts (Cox proportional hazard model, HR: Hazard Ratio, CI: confidence interval).

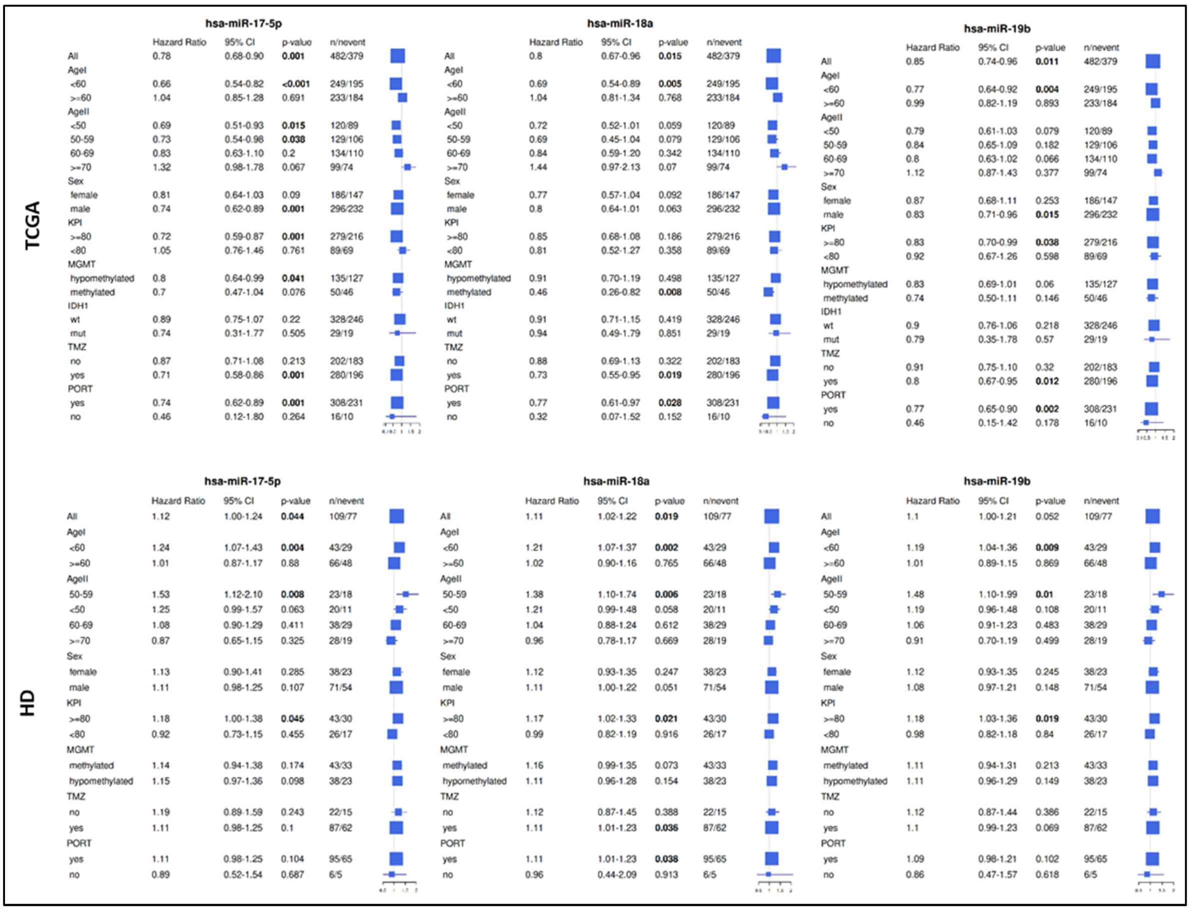

Suppl. Fig. 4: Univariate analysis of miR expression and clinical features in subgroups for miR-17-5p, miR-18a and miR-19b for OS in the TCGA and HD cohort (Cox proportional hazard model, HR: Hazard Ratio, CI: confidence interval).
